# A spatiotemporal state-inference framework for adaptive immunotherapy in glioblastoma

**DOI:** 10.3389/fonc.2026.1875352

**Published:** 2026-06-26

**Authors:** Xiao Chen, Shuping Li, Xiaojun Liu, Wen Ma

**Affiliations:** 1 First Clinical Medical School, Gansu University of Chinese Medicine, Lanzhou, China; 2 Department of Radiotherapy, Gansu Provincial Hospital, Lanzhou, China

**Keywords:** adaptive immunotherapy, glioblastoma, graph neural networks, liquid biopsy, spatiotemporal state inference, tumor microenvironment

## Abstract

Although immunotherapy has transformed outcomes in several solid tumors, it has yielded little survival benefit in glioblastoma (GBM). This limited efficacy may in part reflect not only modest drug activity and a chronically immunosuppressive microenvironment, but also a temporal mismatch between fixed treatment schedules and a tumor–immune ecosystem that evolves across space and time. This review outlines the GBM Immune–Spatiotemporal Feedback Loop (GBM-ISFL), a clinician-governed, hypothesis-generating framework that conceptualizes adaptive immunotherapy as a process of longitudinal sensing, biologic state inference, phase-matched intervention, and iterative feedback. Drawing on single-cell and spatial multi-omics, radiologic assessment, and liquid-biopsy studies, we outline a four-phase atlas of GBM evolution and define a patient-specific Critical Transition Window. This window may functionally overlap with the post-radiotherapy interval highlighted by Response Assessment in Neuro-Oncology (RANO) 2.0, but it should not be treated as a fixed calendar block or as a validated clinical interval. To narrow the resulting observability gap, we position spatiotemporal graph neural networks (STGNNs) as candidate tools for noninvasive inference of latent tumor–immune states from serial multimodal data, including imaging dynamics, treatment exposure, and circulating biomarker trajectories. We further describe how uncertainty-aware state inference could support exploratory phase-specific therapeutic reasoning, translational validation, and lifecycle governance. By reframing GBM immunotherapy around biologic phase rather than chronology alone, the GBM-ISFL offers a testable route toward adaptive, state-informed, and clinically governed precision intervention, but it should not be interpreted as a current standard-of-care algorithm.

## 1 Introduction

Despite maximal safe resection, radiotherapy, and temozolomide-based chemotherapy, glioblastoma (GBM) remains one of the most lethal primary brain tumors in adults. Even with the Stupp protocol, median overall survival is only approximately 15 months, and recurrence is nearly universal. These outcomes suggest that the central problem is not simply a lack of additional therapeutic components, but the continued use of fixed treatment schedules against a biologic system that evolves under surgical, radiotherapeutic, and pharmacologic pressure (1, 2).

Immunotherapy has reshaped the treatment landscape in several solid tumors (3). In GBM, however, phase III studies—including CheckMate-143, CheckMate-498, and CheckMate-548—have not demonstrated a survival benefit (4–7). GBM is often described as immunologically “cold,” but that formulation is incomplete. It does not explain why therapeutic benefit remains limited even when transient inflammatory activity or potentially targetable immune states are present (4).

Several disease-specific barriers help explain why immunotherapy has been less successful in GBM than in other solid tumors (1–4). These include marked spatial and temporal heterogeneity, blood-brain and blood-tumor barrier constraints, variable T-cell infiltration, and myeloid-dominant immunosuppression (8–11). These factors evolve under surgery, radiotherapy, chemotherapy, and salvage treatment pressure rather than acting as static baseline features (8–10, 12), with related evidence from treatment-induced immunoediting and GSC-resistance studies (13, 14). Corticosteroid exposure and treatment-related imaging ambiguity further complicate response assessment (15–17), while molecular modifiers such as MGMT promoter methylation and IDH status affect patient selection and interpretation of therapeutic response (18–21).

Single-cell profiling, spatial multi-omics, and longitudinal imaging support a different interpretation. The GBM tumor microenvironment is not static; it is an evolving ecosystem (8–10, 12). Standard radiochemotherapy can reshape glioma stem cell (GSC) states, reorganize stromal architecture, drive T-cell exhaustion, and reprogram myeloid compartments (10, 13, 14). The key issue, therefore, is not only whether immune suppression exists, but when treatment is delivered relative to that evolution. A therapy given before a resistant program becomes actionable may be premature, whereas the same therapy given after that program has consolidated may be ineffective (9, 10, 13, 14).

Taken together, these observations suggest a timing mismatch between static treatment schedules and the changing biologic phase of the GBM tumor–immune ecosystem (4, 8–10). We hypothesize that a Critical Transition Window (CTW) exists during which the system retains sufficient plasticity for therapeutic redirection before resistant states harden (11, 15, 16). This is a biologically informed, clinically testable hypothesis rather than a validated interval. The 12-week post-radiotherapy period emphasized in Response Assessment in Neuro-Oncology (RANO) 2.0 provides a practical anchor for management and response assessment, but it should not itself be equated with the CTW (11). The window is likely patient specific, heterogeneous, and only partly captured by calendar time (15–17), with clinical and molecular modifiers influencing its boundaries (18–21).

Testing this idea requires near-real-time inference of tumor–immune state, which remains difficult in routine care. Repeated intracranial biopsies are not feasible, and conventional imaging primarily captures macroscopic anatomy rather than the cellular and immunologic processes that drive recurrence. The result is an observability gap between the dynamics clinicians need to act upon and the signals they can realistically measure (10, 22, 23).

Artificial intelligence (AI), particularly spatiotemporal graph neural networks (STGNNs), may help narrow that gap. These models can represent serial multimodal data—MRI-derived radiomics, treatment milestones, liquid-biopsy kinetics, corticosteroid exposure, and clinical status—as evolving relational structures. Used in this way, STGNNs could support noninvasive estimation of latent tumor–immune trajectories over time (24–27). The role of AI here is deliberately limited: it is not a substitute for pathology or clinical judgment, but a candidate state-inference layer that may support uncertainty-aware, phase-matched reasoning (26, 27).

Accordingly, this review presents the GBM Immune–Spatiotemporal Feedback Loop (GBM-ISFL) as a conceptual and translational framework rather than a clinical algorithm. It synthesizes a four-phase atlas of recurrence, examines how STGNN-based state inference might support longitudinal clinical reasoning, outlines phase-specific therapeutic hypotheses, and proposes a translational pathway grounded in biologic plausibility, external validation, uncertainty-aware modeling, and lifecycle governance.

Importantly, the GBM-ISFL should be read as a literature-derived, hypothesis-generating framework rather than a validated clinical algorithm. Established observations include negative checkpoint-inhibitor trials (4–7), GBM heterogeneity and treatment-related imaging ambiguity (9–11), and the practical limits of repeated tissue or systemic sampling (22). Proposed constructs include the CTW, phase-probability estimation, STGNN-based state inference, and phase-matched therapeutic reasoning; all require prospective validation before clinical implementation.

**Figure 1:**
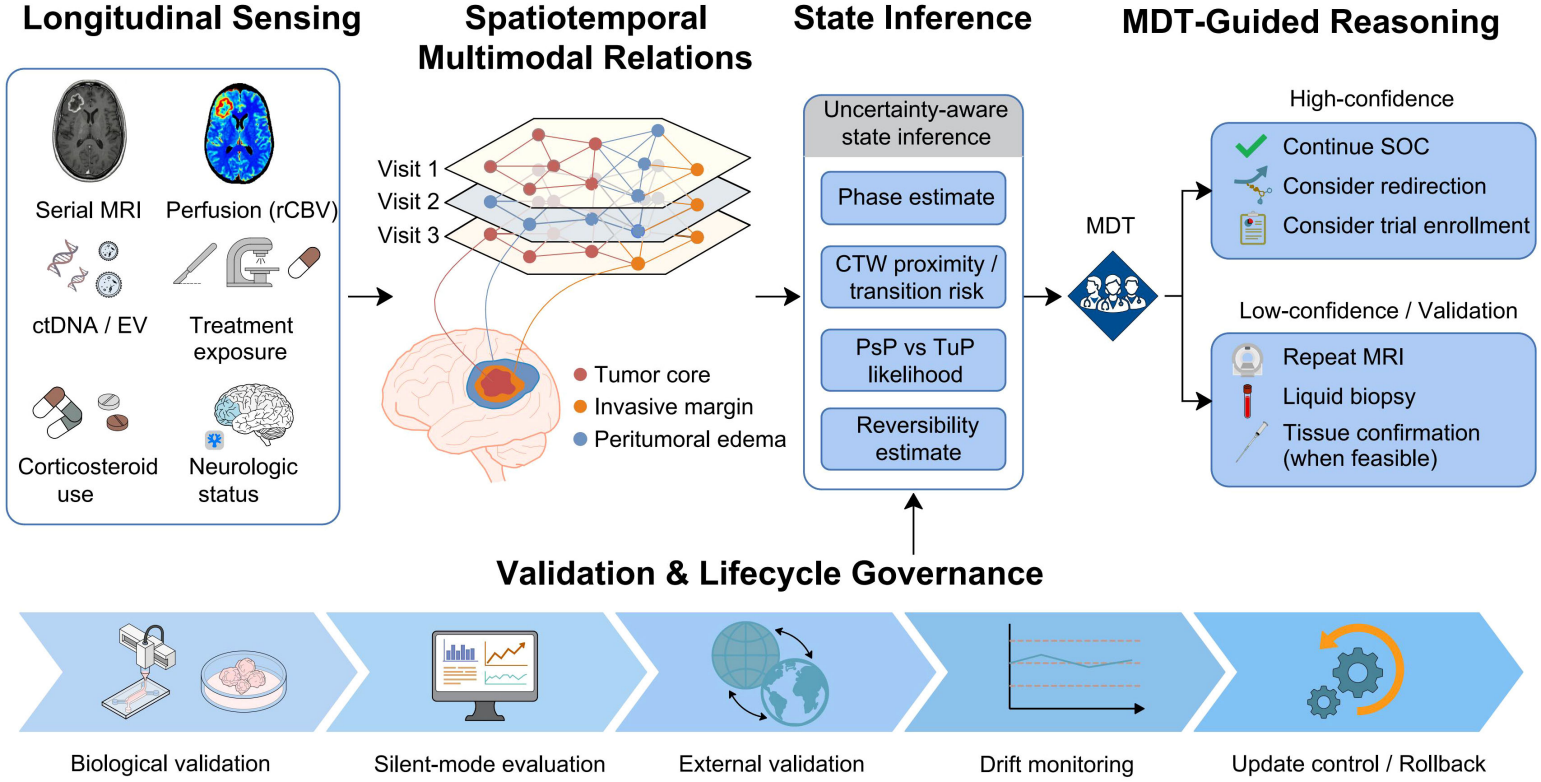
Overview of the GBM-ISFL workflow. Longitudinal multimodal sensing, including serial MRI, perfusion/relative cerebral blood volume (rCBV), circulating tumor DNA (ctDNA) or extracellular vesicle (EV)-associated signals, treatment exposure, corticosteroid use, and neurologic status, feeds a spatiotemporal multimodal relations layer that represents region- and time-aware disease dynamics across serial visits. An uncertainty-aware state-inference layer estimates phase, Critical Transition Window (CTW) proximity and transition risk, pseudoprogression versus true progression (PsP vs TuP) likelihood, and reversibility. Outputs are interpreted through multidisciplinary team (MDT)-guided reasoning. High-confidence outputs may support continuation of standard of care (SOC), consideration of therapeutic redirection, or trial enrollment, whereas low-confidence or validation-requiring outputs trigger repeat MRI, liquid biopsy, or tissue confirmation when feasible. The lower governance ribbon highlights biological validation, silent-mode evaluation, external validation, drift monitoring, and update control/rollback. Abbreviations: GBM-ISFL, GBM Immune–Spatiotemporal Feedback Loop; MRI, magnetic resonance imaging; rCBV, relative cerebral blood volume; ctDNA, circulating tumor DNA; EV, extracellular vesicle; CTW, Critical Transition Window; PsP, pseudoprogression; TuP, true progression; MDT, multidisciplinary team; SOC, standard of care. This diagram is conceptual and does not represent a validated clinical algorithm.

**Figure 1** summarizes the overall GBM-ISFL architecture, and **Table 1** compiles representative clinical and translational evidence that motivates the framework.

**Table 1:** Representative clinical and translational evidence motivating a state-inference framework for adaptive immunotherapy in glioblastoma.

Domain	Representative evidence	Key message	Limitation/failure signal	Relevance to the GBM-ISFL framework
Phase III PD-1 blockade in recurrent GBM	CheckMate-143 (5)	Nivolumab did not improve overall survival over bevacizumab despite a biologically plausible target.	One-time treatment assignment and entrenched immunosuppression likely obscured transiently responsive sub-states.	Supports a shift from static treatment selection toward longitudinal state estimation.
Phase III PD-1 + SOC in MGMT-unmethylated disease	CheckMate-498 (6)	Nivolumab plus radiotherapy did not outperform temozolomide plus radiotherapy.	Immune activation without biologic phase matching may be mistimed, insufficient, or counterproductive in selected contexts.	Reinforces the need for phase-aware stratification before intervention.
Phase III PD-1 + SOC in MGMT-methylated disease	CheckMate-548 (7)	Adding nivolumab to chemoradiotherapy did not improve survival and increased treatment burden.	Poor timing and limited predictive readouts prevented adaptive combination use.	Motivates early noninvasive signals that can justify continuation, withholding, or redirection.
Single-cell and spatial multi-omics (8, 9, 15, 35)	Longitudinal and spatial atlases of the GBM ecosystem	GBM shifts across plastic glioma stem cell, myeloid, and T-cell exhaustion states under treatment pressure.	Biologic transition is spatially heterogeneous and not captured by calendar time alone.	Provides mechanistic support for a four-phase atlas and a patient-specific CTW hypothesis.
AI for PsP versus TuP discrimination (33, 34, 77)	Multimodal MRI/machine-learning studies and meta-analyses	Performance is promising, but heterogeneity and gaps in external validation remain substantial.	Prediction confidence, transportability, and workflow integration remain limiting.	Justifies uncertainty-aware, clinician-facing deployment rather than autonomous use.
Liquid-biopsy monitoring (22, 68–70)	CSF-enriched ctDNA/EV studies	Dynamic biomarkers may capture biologic time and emerging resistance earlier than MRI alone.	Sampling feasibility, assay sensitivity, and longitudinal standardization remain limiting.	Supports multimodal longitudinal sensing rather than image-only inference.

GBM, glioblastoma; PD-1, programmed cell death protein 1; SOC, standard of care; MGMT, O^6^-methylguanine-DNA methyltransferase; CTW, Critical Transition Window; PsP, pseudoprogression; TuP, true progression; MRI, magnetic resonance imaging; ctDNA, circulating tumor DNA; EV, extracellular vesicle; CSF, cerebrospinal fluid.

## 2 Spatiotemporal evolution of the GBM ecosystem: a state-space atlas for phase-aware clinical reasoning

To operationalize this premise, we organize GBM recurrence into four biologically defined phases: Early, Mid, Late, and Ultra-late. This atlas is not intended as a rigid calendar taxonomy. Rather, it is a state-space framework that links clinically observable events to coordinated shifts in GSC identity, immune composition, stromal structure, and metabolic constraint (15, 16, 28, 29).

Conventional timepoints—postoperative days, the end of radiotherapy, and scheduled MRI visits—remain useful, but only as weak temporal priors (17, 23). The more relevant axis is biological time, which must be inferred from changing observables such as contrast-enhancement kinetics, perfusion trends, corticosteroid requirement, neurologic status, and liquid-biopsy trajectories, including circulating tumor DNA (ctDNA) or extracellular-vesicle signals (30–32). On this view, phase transitions are probabilistic state shifts rather than fixed calendar dates (27, 32).

A central element of the atlas is the Critical Transition Window. We define it as a metastable interval during which the tumor–immune ecosystem remains at least partly reversible before exhaustion locking, stromal consolidation, and metabolic constraint become firmly established (19–21). This interval may functionally overlap with the post-radiotherapy period highlighted by RANO 2.0, but the two should not be treated as identical in every patient (11). RANO 2.0 offers a practical clinical anchor; the CTW, by contrast, represents a biologically individualized transition state whose timing may vary across patients and align only imperfectly with calendar time (11, 15–17).

Operationally, the CTW should be treated as a probabilistic transition state rather than a binary or calendar-defined interval. In a future clinical workflow, CTW proximity would require convergence across longitudinal signals, including enhancement kinetics, perfusion or rCBV trends, corticosteroid requirement, neurologic trajectory, and treatment exposure (11, 17, 33, 34). When available, ctDNA or EV-associated tumor-signal kinetics could provide an additional systemic layer (22, 23, 30, 32). No single marker should be considered sufficient to define CTW entry or exit; RANO 2.0 provides a clinical assessment anchor, not a surrogate definition of the CTW itself (11).

The four phases described below should therefore be interpreted as operating regions rather than fixed bins. The Early Phase captures acute inflammatory remodeling and tumor co-option. The Mid Phase centers on the CTW and progressive narrowing of reversibility. The Late Phase reflects exhaustion locking together with metabolic constraint, whereas the Ultra-late Phase marks a shift toward immune-desert conditions and tumor-intrinsic dependencies (9, 15).

Similarly, the four-phase atlas is not intended to compress GBM evolution into a universal linear sequence. Phase assignment should be probabilistic and conditional on patient- and tumor-level modifiers, including IDH status, MGMT promoter methylation, extent of resection, corticosteroid exposure, treatment history, and recurrence pattern (11, 18, 19, 21). Tumor location and spatial niche composition further shape phase inference (9, 14, 35). Patients may not pass through these phases uniformly, and different tumor regions may occupy different biological states at the same clinical timepoint.

**Figure 2:**
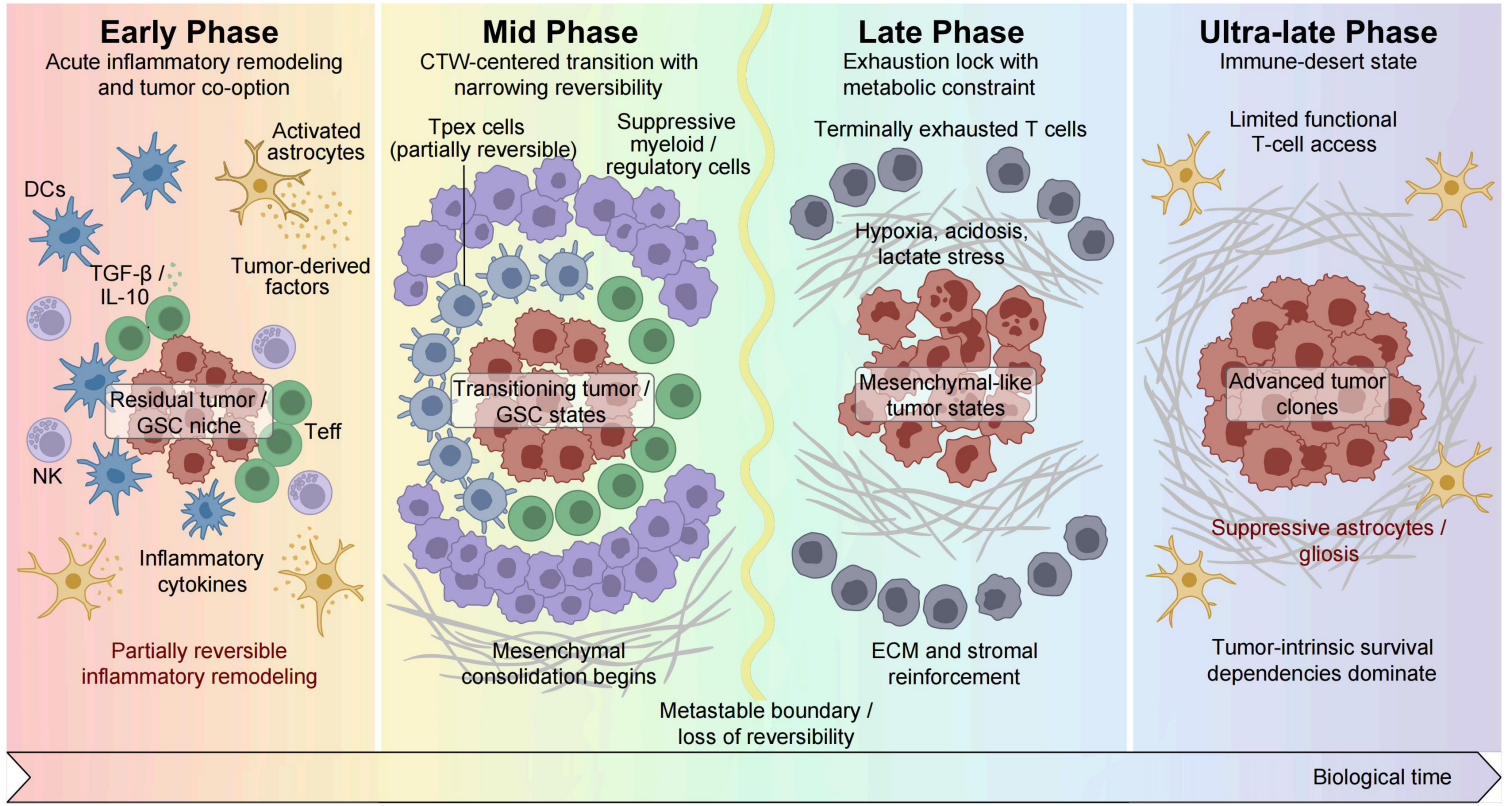
Four-phase atlas of the GBM tumor–immune ecosystem across biological time. The early phase is characterized by acute inflammatory remodeling and tumor co-option, with dendritic cells, natural killer cells, effector T cells, inflammatory cytokines, activated astrocytes, tumor-derived factors, and a residual tumor/glioma stem cell (GSC) niche contributing to a partially reversible inflammatory state. The mid phase represents a CTW-centered transition in which Tpex cells, suppressive myeloid/regulatory cells, transitioning tumor/GSC states, and emerging mesenchymal consolidation coexist while reversibility narrows. The late phase reflects exhaustion lock with metabolic constraint, including terminally exhausted T cells, hypoxia/acidosis/lactate stress, mesenchymal-like tumor states, and extracellular matrix (ECM) and stromal reinforcement. The ultra-late phase approaches an immune-desert state with limited functional T-cell access, advanced tumor clones, suppressive astrocytes/gliosis, and tumor-intrinsic survival dependencies. The wavy boundary marks a metastable transition with progressive loss of reversibility rather than a fixed calendar transition. Abbreviations: CTW, Critical Transition Window; DCs, dendritic cells; NK, natural killer cells; Teff, effector T cells; GSC, glioma stem cell; Tpex, precursor/progenitor exhausted T cells; ECM, extracellular matrix; IL-10, interleukin-10; TGF-β, transforming growth factor-β. The atlas is a schematic state-space heuristic rather than a rigid linear staging system.

**Figure 2** provides a schematic overview of this four-phase atlas, including the CTW and the progressive narrowing of reversibility across biological time.

### 2.1 Early phase: acute inflammation and tumor co-option

The Early Phase spans the period after resection and through early radiochemotherapy, when tissue injury and immunogenic cell death generate a transient inflammatory response (36, 37). Damage-associated molecular patterns can recruit innate immune cells into the tumor bed and briefly increase antigen exposure. That signal is unstable, however. Residual GSC populations can exploit cytokine-rich environments through pathways that include IL-6, TNF-α, NF-κB, and STAT3, thereby driving adaptive state transitions such as proneural-to-mesenchymal reprogramming (37–40).

At the same time, tumor-infiltrating T cells may still retain a precursor/progenitor exhausted T cell phenotype, with residual proliferative capacity and dysfunction that is not yet fixed (41, 42). Unstable GSC identity and incompletely exhausted immune states thus coexist, creating a transiently reversible configuration (13, 41, 42).

This phase is highly labile. Potential readouts include evolving peritumoral edema, early heterogeneity of contrast enhancement, perfusion shifts, and emerging liquid-biopsy signals. The key biologic question is whether inflammatory remodeling can still be redirected or whether it has already begun to consolidate into a mesenchymal, myeloid-dominant niche (23, 30).

### 2.2 Mid phase: CTW-centered transition

The Mid Phase marks the transition between transient immune-mediated control and consolidated immune dysfunction (11, 15, 16). It is also the interval in which pseudoprogression and true progression may be most difficult to distinguish, even though some biologic reversibility may still remain (33, 34).

Two processes are especially important in this interval. First, GSC populations may stabilize epigenetically toward mesenchymal-like, immune-evasive phenotypes, thereby reducing antigen visibility and strengthening stromal protection (13, 14). Second, ongoing antigen exposure and inhibitory signaling can drive T cells from Tpex-like states toward terminally exhausted (Tex) states, with progressive loss of proliferative capacity and effector function (41–44).

A potentially informative systems-level signal is the balance between suppressive myeloid cells and effector lymphocytes, particularly at the invasive margin. A rising ratio of M2-polarized tumor-associated macrophages (TAMs) to effector T cells may indicate that the window is closing. Once this shift consolidates, reversibility is likely to decline sharply (45, 46).

The practical challenge is to determine whether a patient is approaching, entering, or leaving this transition. Serial imaging dynamics, including contrast-enhancement velocity or relative cerebral blood volume (rCBV), combined with liquid-biopsy kinetics may improve discrimination between pseudoprogression and true progression and may also help estimate transition probability (23, 33, 34). This may represent the last interval in which suppressive programs can still be destabilized before they harden. Once missed, the system is more likely to progress into a low-plasticity state characterized by exhaustion locking (4, 43, 44) and metabolic constraint (47–50).

Two simplified examples illustrate the intended use of this concept. Increased enhancement within the RANO-relevant post-radiotherapy interval, stable neurologic status, decreasing corticosteroid requirement, and non-progressive rCBV would favor treatment-related change over irreversible CTW exit (11, 17, 33, 34). Stable or declining ctDNA/EV signal would further support this interpretation when available (22, 23, 30, 32). In contrast, increasing enhancement velocity, rising rCBV, worsening neurologic symptoms, increasing corticosteroid requirement, and persistent or rebounding circulating tumor signals would increase concern for true progression and declining reprogrammability. These examples clarify clinical reasoning and should not be read as treatment rules.

### 2.3 Late phase: exhaustion lock and metabolic constraint

By the Late Phase, the tumor–immune ecosystem has lost substantial plasticity and is more difficult to perturb. T cells increasingly display terminal exhaustion programs, including TOX^high^ phenotypes and weak responsiveness to checkpoint blockade. The dominant problem is no longer transient suppression alone, but exhaustion locking reinforced by epigenetic stabilization (4, 41, 47).

In parallel, mesenchymal-like GSC populations help create a metabolically hostile niche marked by hypoxia, acidosis, altered nutrient availability, and competition for key substrates (48–51). These conditions act together with immunosuppressive myeloid populations, including TGF-β-producing TAMs, to generate both biochemical and physical barriers to effective immune function (46, 49–51).

Because metabolic and epigenetic restraints reinforce one another, isolated immune activation is unlikely to be sufficient at this stage. A more plausible objective is coordinated reprogramming—an “unlock and refuel” strategy that couples partial reversal of dysfunctional immune programs with restoration of metabolic conditions more compatible with antitumor activity (50, 52–54).

At this stage, the critical issue is not merely phase assignment, but whether meaningful reversibility remains and which intervention combinations remain biologically credible.

### 2.4 Ultra-late phase: immune desert state and target reprogramming

The Ultra-late Phase reflects a deeper regime shift in which tumor progression becomes less dependent on active immune interaction and more dependent on tumor-intrinsic survival programs (14, 55, 56). Functional lymphocyte infiltration is sparse, and the ecosystem approaches an immune-desert state (47, 56).

Advanced GSC clones in this setting may acquire phase-specific dependencies, including greater reliance on oxidative phosphorylation, fatty-acid metabolism, stress-response programs, or epigenetic regulators such as EZH2 (48–50, 57). Some of these liabilities may be far less apparent earlier in disease evolution, underscoring that the therapeutic landscape itself changes with phase.

At this stage, emphasis may shift from restoring endogenous immunity to exploiting tumor-intrinsic vulnerabilities. Synthetic lethal targeting, target-discovery workflows, and precision-editing concepts (58–61), together with next-generation cellular therapies (62, 63), may therefore become more relevant than conventional immunomodulation alone.

The priority in this phase is vulnerability discovery rather than finer immune-phase labeling. Longitudinal treatment history, imaging trajectories, molecular context, and prior response patterns may help prioritize latent dependencies for experimental or clinical testing (26, 59, 64). The term “immune desert” should not be interpreted as complete absence of immune cells; rather, it denotes a state in which effective infiltration, accessibility, or antitumor function has markedly diminished (50, 64).

### 2.5 Evidence for a critical transition window

Support for a Critical Transition Window comes from convergent observations across molecular, spatial, radiologic, and systemic domains. Even so, the CTW should still be regarded as a working biologic hypothesis rather than a universally fixed interval. The common signal across these lines of evidence is that loss of therapeutic reversibility in GBM appears to unfold over a finite metastable period rather than in a single step. During that interval, tumor and immune programs remain dynamically coupled and at least partly redirectable (9, 11, 15, 16).

At the molecular level, longitudinal single-cell and multi-omics studies point to progressive rather than abrupt state transitions (8, 12, 65). Malignant GBM cells move across plastic cellular states under therapeutic and microenvironmental pressure, while CD8-positive T-cell populations appear to shift from progenitor-like or precursor-exhausted states toward terminally exhausted states with reduced proliferative capacity and diminished responsiveness to rescue (8, 12, 42, 43). This pattern supports the existence of a finite interval during which immune plasticity may still be sufficient for therapeutic redirection before exhaustion programs become epigenetically fixed (41–43, 47).

This transition is unlikely to occur uniformly across the lesion. Spatial multi-omics studies suggest that hypoxic, necrotic, vascular, and invasive-margin niches form distinct but interacting compartments that shape both tumor-state plasticity and immune suppression. Mesenchymal transition, myeloid remodeling, and immune dysfunction may therefore consolidate in a spatially staggered manner rather than all at once, a pattern more consistent with a metastable systems state than with a single binary event (15, 35).

Imaging reflects the same problem from another perspective. Early post-radiotherapy studies are notoriously difficult to interpret (10, 33, 66). RANO 2.0 treats the first 12 weeks after radiotherapy as a decision-sensitive interval in which treatment-related change can mimic progression. That does not mean that the CTW is identical to the RANO 2.0 interval (11). Rather, the 12-week period provides a practical clinical anchor within which biologic transition is especially likely to be encountered (11, 17). Advanced imaging markers, including perfusion measures such as relative cerebral blood volume, may add biologic specificity during this period and provide useful surrogate signals for state inference (10, 17, 66).

Liquid-biopsy kinetics provide a second systemic layer of evidence. These biomarkers remain technically challenging in GBM and are not ready to guide routine care on their own. Even so, early decreases in ctDNA or extracellular-vesicle-associated tumor signals may align with durable control, whereas persistent or rebounding signals may foreshadow radiographic progression. Dynamic biomarker trajectories may therefore track biological time more closely than calendar time alone, especially when interpreted alongside serial imaging and treatment exposure (67–70).

None of these observations proves the existence of a universally definable transition window. Taken together, however, they support the plausibility of a patient-specific metastable interval during which the tumor–immune ecosystem remains partly reversible (9, 15). In this framework, the relevant threshold is not certainty, but sufficient evidence to justify state-aware longitudinal monitoring and hypothesis-driven testing rather than routine clinical action (15, 27).

## 3 STGNN-based longitudinal state inference: a candidate clinical decision-support layer

Once the atlas is defined, the next challenge is observability. The biologic variables most relevant to treatment timing in GBM—GSC plasticity, T-cell exhaustion trajectories, myeloid reprogramming, and metabolic locking—cannot be tracked directly through repeated intracranial sampling in routine practice.

This creates a gap between the disease dynamics clinicians need to understand and the signals they can actually measure. Repeated tissue acquisition is impractical, and conventional imaging mainly captures anatomy and treatment-related change rather than the cellular and immunologic processes that drive recurrence. Adaptive intervention therefore cannot depend on direct observation alone; it requires a computational surrogate capable of inferring latent tumor–immune states from longitudinal, noninvasive, clinically obtainable data (10, 22, 23).

We propose spatiotemporal graph neural networks (STGNNs) as one candidate state-inference system. The aim is not to replace pathology, but to integrate serial imaging, treatment milestones, corticosteroid exposure, and liquid-biopsy kinetics within a common spatiotemporal structure and to use that structure to estimate biologic phase, transition risk, pseudoprogression probability, and therapeutic reversibility under explicit uncertainty constraints (27, 70, 71).

### 3.1 Why STGNNs? modeling a relational and dynamic ecosystem

STGNNs are well suited to this problem for two reasons: the biology is relational, and the clinic is irregular. Tumor cells, immune populations, vascular niches, edema, necrosis, and invasive margins evolve as coupled components of a single ecosystem. Clinical follow-up is similarly uneven: imaging intervals vary, treatment changes are asynchronous, and different modalities are often missing at different visits. A useful inference framework must accommodate both features simultaneously (25, 35, 72).

More conventional architectures capture only parts of this structure. Convolutional neural networks are strong at local spatial pattern recognition, but they do not naturally encode longitudinal dependency, cross-modal interaction, or irregular follow-up. Recurrent models handle temporal order better, yet they often assume more sequential regularity than real neuro-oncology data provide and do not explicitly model spatial relationships among tumor regions or across modalities. STGNNs are therefore better matched to partially observed, non-Euclidean, longitudinal clinical data (24–26).

This distinction matters in real-world neuro-oncology, where missing modalities, skipped visits, corticosteroid-related confounding, and heterogeneous acquisition protocols are common (27, 73, 74). A graph representation can absorb incomplete observations without forcing them onto an artificial regular grid. Nodes may represent anatomical regions, timepoints, or region–time composites, and edges may encode spatial adjacency, temporal continuity, and cross-modal dependence (24, 26, 27). Through message passing, information from neighboring regions, adjacent visits, or alternate modalities can partially compensate for missing inputs and help preserve continuity of inference under sparse clinical conditions (64, 73, 74).

### 3.2 Cross-modal state reconstruction: inferring the invisible

The ambition here is not merely to classify imaging appearances, but to reconstruct latent biologic states from clinically observable data (26, 70, 71, 75). In practical terms, that means estimating hidden variables—GSC plasticity, T-cell exhaustion trajectories, myeloid remodeling, and metabolic constraint—from longitudinal signals that clinicians can actually obtain (22, 23, 75).

MRI-derived radiomics can provide proxy information on cellularity, vascularity, permeability, edema, necrosis, and invasive behavior. Liquid-biopsy trajectories can capture tumor burden, treatment response, or clonal evolution at a systemic level (68–71). Treatment milestones, corticosteroid exposure, neurologic status, and prior response patterns further shape the interpretation of imaging change (23, 30). No single modality is sufficient: each captures only part of the process and can mislead when interpreted in isolation (68–70). Considered together over time, however, these signals offer a more faithful picture of the evolving tumor–immune state (68–71).

In an STGNN, these heterogeneous inputs can be represented within a unified spatiotemporal graph in which spatial regions, clinical visits, biomarkers, and treatment events all contribute relational information to a shared latent representation. The goal is not to claim direct visualization of immune-cell composition or tumor-cell state, but to estimate the probability distribution of hidden variables given the available evidence (24–27). The latent state vector should therefore be interpreted as a clinically useful surrogate of biologic phase rather than as a one-to-one molecular readout (24–27).

A future STGNN implementation would require a prespecified graph schema. Nodes could represent enhancing tumor, non-enhancing tumor, peritumoral edema, necrotic compartments, invasive margins, clinical visits, circulating biomarker measurements, and treatment events. Edges could encode spatial adjacency, temporal continuity, cross-modal dependence, and treatment-to-state relationships (24–27). Candidate input features would include contrast-enhanced T1, T2/FLAIR, diffusion/ADC, perfusion or rCBV trends, radiomic delta features, corticosteroid exposure, neurologic status, treatment milestones, MGMT and IDH status, extent of resection, and ctDNA or EV kinetics when available (64, 74, 76).

Training and validation targets should be prespecified and clinically meaningful, including pseudoprogression versus true progression, time to progression, progression-free survival, overall survival, biopsy-confirmed recurrence when feasible, CTW-proximity calibration, and reprogrammability estimates (33, 34). Missing data should be addressed with modality masking, time-aware imputation, graph message passing under missing modalities, and sensitivity analyses (24–27). Performance should be benchmarked against radiomics-only models, CNN/RNN/Transformer architectures, and RANO-based clinical heuristics, with explicit evaluation of calibration, missing-data robustness, decision-curve utility, and external transportability (74, 77–79), alongside prospective evaluation planning (80).

To be clinically useful, outputs must map onto decisions that clinicians already face. Relevant examples include phase probabilities, a transition-risk score reflecting likely entry into or exit from the CTW, pseudoprogression-versus-true-progression probabilities, and a reprogrammability score that captures the likelihood that a suppressed state remains biologically reversible. Framed in this way, the latent state vector becomes a decision-oriented summary of where the tumor–immune ecosystem most likely lies and how it is evolving (33, 34, 77).

This is why multimodal longitudinal inference is hypothesized to outperform single-modality prediction and should be tested against radiomics-only, CNN/RNN/Transformer-based, and RANO-based clinical baselines. An increase in enhancement may suggest progression under one set of biomarker and clinical conditions, but treatment-related remodeling under another. Likewise, a stable imaging pattern may carry different implications depending on perfusion trends, corticosteroid requirement, neurologic change, and circulating tumor-signal kinetics (22, 23, 33, 34).

### 3.3 Trust layer: uncertainty, interpretability, and safety constraints

For a clinically deployed inference system, predictive performance alone is not enough. Trust must be built into model development, validation, and use (74, 78, 79).

First, outputs should be uncertainty-aware. Deep ensembles, Monte Carlo dropout, and conformal prediction can all be used to generate calibrated confidence estimates for major outputs. When uncertainty exceeds predefined thresholds, the appropriate response is not automated action, but a hold-for-validation state that triggers additional assessment, such as repeat imaging, liquid biopsy, or tissue confirmation when feasible (78, 79, 81).

Second, predictions must be interpretable at a level clinicians can meaningfully evaluate. Attention maps or attribution summaries can indicate which spatial regions, time intervals, or input modalities most influenced a prediction (24, 64, 79). The standard is not perfect causal explanation, but sufficient transparency for clinicians to judge whether the model is relying on biologically plausible patterns rather than noise or artifact (64, 79).

Third, validation must extend beyond internal performance (74, 80). External testing across multicenter cohorts, with locked parameters and prespecified endpoints, is essential if the model is to claim generalizability (26, 64, 74). Prospective silent-mode studies—where predictions are generated but not used to guide treatment—provide a necessary bridge between retrospective development and interventional use (64, 74, 80).

Final interpretation must remain with the physician and the multidisciplinary team. These models are intended to support clinical reasoning rather than replace it, especially when predictions conflict with neurologic status, pathology, corticosteroid dependence, or other elements of the broader clinical picture (64, 79).

### 3.4 Dynamic decision support: from prediction to clinical reasoning

In this setting, the output of an STGNN is not a static label, but a dynamic decision-support signal. At each assessment point, the model updates its estimate of current phase, near-term trajectory, and associated uncertainty. Those estimates then need to be interpreted in light of the specific clinical question facing the team (24, 64).

During the Mid Phase, for example, the most immediate application may be discrimination between pseudoprogression and true progression at RANO-relevant decision points (11, 17, 22, 77). If the model favors pseudoprogression and biomarker trends are stable or improving while clinical status remains acceptable, continuing the current regimen may be reasonable (22, 23, 33, 34). If the trajectory instead supports true progression, rising transition risk, and falling reversibility, earlier escalation, trial referral, or therapeutic redirection may warrant consideration (33, 34, 77).

More broadly, the state vector can function as a structured aid for phase-matched intervention (64). Forecasted movement toward exhaustion locking may prompt consideration of reprogramming strategies. Inference of an immune-desert state may shift attention toward tumor-intrinsic vulnerabilities (45, 50, 82, 83). In every case, model outputs should inform multidisciplinary judgment rather than substitute for it (64).

Dynamic decision support therefore involves more than assigning diagnostic probabilities. It translates longitudinal inference into time-sensitive clinical reasoning, allowing intervention to be adjusted to where the system is, where it appears to be moving, and how certain that estimate is at the moment of action.

### 3.5 From model development to clinical deployment

A credible STGNN program must be developed in stages. Early work should establish analytic validity through temporal-split validation, robustness testing across scanners and institutions, and ablation studies that demonstrate the value of multimodal integration. External validation should then assess performance in geographically and temporally distinct cohorts (26, 64, 74, 84).

Only after these steps should prospective silent-mode evaluation begin, to test workflow fit and concordance with multidisciplinary tumor board reasoning. Clinician-facing deployment should follow only if performance, calibration, interpretability, and safety thresholds are all satisfied (64, 74, 80).

Deployment is not the end of the process. The system must remain under lifecycle governance. Dataset shift, performance drift, and unintended bias all require monitoring, and model updates should occur only within predefined and validated boundaries (74, 79, 85). In this sense, the STGNN operationalizes the GBM-ISFL as a governed clinical state-inference system rather than an autonomous actor (79, 85, 86).

## 4 Phase-matched therapeutic reasoning in GBM: translating state inference into adaptive clinical hypotheses

Within the GBM-ISFL, therapeutic reasoning is anchored to inferred biologic state rather than to a fixed calendar sequence. This is where state inference is most clinically relevant: it may help identify when inflammation should be redirected rather than amplified, when a partly reversible transition might still be salvaged, when exhaustion and metabolic constraint call for coordinated reprogramming, and when therapeutic logic should shift from immune rescue to tumor-intrinsic vulnerabilities (64).

The latent state vector derived from longitudinal multimodal data is intended to function as a structured clinical signal rather than an abstract computational artifact. By integrating serial imaging, liquid-biopsy kinetics, treatment exposure, corticosteroid requirement, and neurologic status, the model may help estimate current phase, near-term trajectory, and the likelihood that meaningful reversibility remains (22, 23, 26, 64). These outputs do not prescribe treatment on their own. Instead, they help clinicians identify which biologic bottleneck is most likely dominant at a given timepoint and which intervention logic may therefore be most appropriate.

The phase-specific strategies below are hypothesis-driven therapeutic logics rather than standards of care. Their purpose is to show how longitudinal state inference could be translated into phase-matched therapeutic reasoning across the Early, Mid, Late, and Ultra-late phases, with treatment aligned to the evolving tumor–immune state rather than assumed to carry the same meaning throughout the course of disease (4, 64).

Accordingly, references to myeloid blockade, TGF-β modulation, metabolic reconditioning, epigenetic therapy, cellular therapy, or oncolytic approaches should be read as trial-oriented biological hypotheses rather than treatment recommendations. Their inclusion in the framework reflects plausible phase-dependent bottlenecks, not evidence that these interventions should currently be assigned to individual GBM patients on the basis of inferred phase.

**Table 2** summarizes this phase-matched reasoning framework across Early, Mid (CTW-centered), Late, and Ultra-late states.

**Table 2:** Hypothesis-oriented phase-matched reasoning matrix for the GBM-ISFL framework.

Phase	Dominant ecosystem state	Practical readout focus	Primary clinical question	Action logic and guardrails
Early	Inflammatory remodeling, residual GSC plasticity, and partially plastic T-cell states.	Postoperative MRI, perfusion, steroid exposure, and early liquid-biopsy signals when feasible.	Is acute inflammation being redirected toward durable antitumor immunity or toward suppressive niche formation?	Redirect inflammation and block plasticity-driving or myeloid-recruiting cues; outputs should remain decision-support only at this stage.
Mid (CTW-centered)	Mesenchymal consolidation begins; Tpex-to-Tex transition emerges; myeloid dominance rises while partial reversibility persists.	Serial MRI/rCBV, contrast-enhancement velocity, and ctDNA or EV trajectories.	Is the patient entering or exiting the CTW, and is the observed change more consistent with PsP or TuP?	Continue current management with short-interval confirmation if PsP is likely; redirect or salvage if TuP is more likely while reversibility persists. Use decision support only when confidence is adequate; otherwise repeat imaging, MDT review, or biopsy.
Late	Exhaustion lock, hypoxia/acidosis, metabolic constraint, and stromal reinforcement.	Longitudinal delta features, worsening perfusion or metabolic proxies, and declining reversibility scores.	Can the current state still be reprogrammed, or has meaningful plasticity largely been lost?	Combine immune, epigenetic, and metabolic reconditioning; prefer MDT-supervised or trial-oriented use, because checkpoint escalation alone is unlikely to suffice.
Ultra-late	Immune-desert conditions with increasing tumor-intrinsic survival dependencies.	Treatment history, advanced imaging context, and re-biopsy/genomic profiling when feasible.	Should strategy shift away from immune rescue toward dependency-targeted or experimental therapy?	Pivot toward dependency-targeted or experimental strategies (e.g., synthetic lethality, next-generation cell therapy, or engineered oncolytic approaches) with strict human-in-the-loop governance.

CTW, Critical Transition Window; GSC, glioma stem cell; Tpex, precursor/progenitor exhausted T cell; Tex, terminally exhausted T cell; MRI, magnetic resonance imaging; rCBV, relative cerebral blood volume; ctDNA, circulating tumor DNA; EV, extracellular vesicle; PsP, pseudoprogression; TuP, true progression; MDT, multidisciplinary team.

### 4.1 Early phase: redirecting inflammation

Immediately after resection and during early radiotherapy, the tumor microenvironment is dominated by tissue injury, cytokine flux, and unstable GSC adaptation. Although this state may appear transiently immunologically favorable, it is also highly susceptible to tumor co-option. The primary therapeutic objective in this phase is therefore not maximal immune stimulation, but prevention of the conversion of wound-healing and inflammatory signals into durable mesenchymal plasticity, suppressive myeloid recruitment, and downstream immune escape (36–39).

In this setting, interventions directed at pathways such as NF-κB or STAT3 could be prioritized for experimental evaluation when they uncouple inflammatory cues from tumor-promoting state transitions (37, 39). Modulation of chemokine axes involved in suppressive myeloid recruitment, particularly CCL2–CCR2, may also help preserve a more permissive immune environment for later therapy (87–89). The practical value of state inference here lies in determining whether acute inflammatory remodeling remains potentially productive or has already begun to organize a suppressive niche. The objective is redirection rather than indiscriminate amplification. If this opportunity is missed, the system is more likely to enter the Mid Phase with less residual reversibility (13, 37–39).

### 4.2 Mid phase: navigating the critical transition window

The Mid Phase may be the most decision-sensitive interval in the framework. Treatment-related remodeling and genuine tumor regrowth can be difficult to distinguish, yet some reversibility may still remain (11, 17, 33, 34). Clinically, this interval may overlap with the post-radiotherapy period emphasized in RANO 2.0, but within the GBM-ISFL it is treated as a patient-specific transition state rather than a fixed calendar block (11, 17, 33, 34).

The immediate clinical question is whether the observed trajectory is more consistent with pseudoprogression or true progression. When longitudinal imaging dynamics, perfusion trends, and biomarker trajectories favor pseudoprogression, continuation of the current regimen may help preserve a useful immune response and avoid premature treatment switching. When the inferred trajectory instead supports true progression—especially in the setting of mesenchymal transition, increasing myeloid dominance, or declining reprogrammability—earlier therapeutic redirection may warrant consideration (33, 34, 77).

In this phase, strategies targeting colony-stimulating factor 1 receptor (CSF1R)-dependent myeloid programs, TGF-β-mediated stromal signaling, or related suppressive circuits could be prioritized for experimental evaluation before exhaustion and stromal consolidation become fully entrenched (82, 90–92). The rationale is not that any single regimen is uniquely indicated, but that this may represent the last biologically plausible interval either to preserve residual plasticity or to attempt reopening it before dysfunction becomes more durable. After the window closes, therapeutic logic shifts toward reprogramming a more constrained, low-plasticity ecosystem (90–92).

### 4.3 Late phase: reprogramming a locked system

Once the transition window narrows or closes, therapeutic logic changes substantially. In the Late Phase, resistance reflects both immune-cell state and tumor-niche metabolism: T-cell dysfunction becomes increasingly stabilized, the surrounding microenvironment becomes hypoxic, acidic, and nutritionally competitive, and checkpoint escalation alone is unlikely to provide durable benefit (43, 44, 54).

A more biologically plausible strategy at this stage is coordinated reprogramming. Epigenetic interventions may partially restore responsiveness in exhausted T cells or destabilize suppressive transcriptional programs. Metabolic approaches targeting tryptophan catabolism, glutamine dependence, hypoxia adaptation, or lactate handling may help recondition the niche to better support antitumor immunity. The goal is not simply to intensify immune stimulation and hope for rescue, but to loosen the interacting barriers that maintain dysfunction (50, 52–54).

At this stage, state inference shifts from transition detection toward estimation of reprogrammability—whether meaningful reversibility still exists and which combinatorial leverage points remain biologically plausible in a given patient. If reversibility has largely been lost, strategy may need to move beyond immune rescue toward target switching based on tumor-intrinsic dependencies (43, 52–54).

### 4.4 Ultra-late phase: target switching

With further progression, the central question may no longer be how to rescue endogenous immunity, but whether that goal remains biologically realistic at all. In the Ultra-late Phase, the ecosystem approaches an immune-desert state: effective lymphocyte engagement is sparse, and tumor progression depends increasingly on intrinsic metabolic, epigenetic, and stress-adaptation programs. Conventional immunomodulation alone is therefore unlikely to provide substantial benefit (47, 55, 56).

Therapeutic attention may therefore need to shift toward vulnerabilities that emerge in this advanced context, including dependence on oxidative phosphorylation, fatty-acid metabolism, chromatin regulators such as EZH2, or liabilities created by prior therapy and selection pressure (50, 57). Cellular therapies, engineered oncolytic approaches, or synthetic lethal strategies may be more relevant if they can bypass, rather than merely reverse, the local immune deficit (62, 63, 93). In this setting, the role of computational inference is less to rescue a failing immune response than to help prioritize which tumor-intrinsic liabilities may still be actionable (50, 62, 63, 93).

Even so, these options remain exploratory. The value of the framework is to organize how such vulnerabilities might be prioritized, not to imply that they are already validated for routine use.

Across all phases, adaptation should be iterative. Each intervention generates new imaging, molecular, and clinical information, and those data can be folded back into the state-inference system to update estimates of phase, trajectory, and residual reversibility. The result is not a series of isolated choices but a continuing prediction–intervention–feedback cycle, which in turn motivates the translational pathway discussed next (74, 94–96).

## 5 Translational pathway: from computational inference to clinically governed adaptive systems

For the GBM-ISFL to move beyond a conceptual model, translation must connect computational inference with biologic plausibility, clinical validity, and regulated implementation. Model-derived hypotheses should be tested in experimental systems, compared against longitudinal patient trajectories, and constrained by governance standards before they influence care (26, 74, 85, 97).

### 5.1 Biological validation: from prediction to plausibility

A core requirement is to show that model-inferred states correspond, at least in part, to biologically observable phenomena. Because STGNN outputs are latent and probabilistic, credibility depends on triangulation rather than on one-to-one proof (26, 97, 98).

Patient-derived organoids, immune co-culture systems, microfluidic GBM-on-a-chip platforms, and spatially resolved three-dimensional models can each contribute to this validation ecosystem. They preserve different aspects of native tumor architecture, immune interaction, vascular transport, and hypoxic stress. Used together, they allow investigators to test whether model-guided hypotheses—for example, that a given state should respond to myeloid blockade, metabolic reconditioning, or phase-matched intervention—produce the expected directional biologic effects (97–100), with microfluidic and three-dimensional platforms providing complementary support (101, 102). Recent reviews of human and tumor organoid systems further support their value as intermediate validation platforms for disease modeling, drug testing, and translational decision support, although their ability to reproduce the full immune, vascular, and spatial complexity of GBM remains incomplete (103, 104). Broader work on treatment-modifiable cancer-cell properties also supports the need to view tumor state as a multidimensional and dynamically regulatable construct rather than as a static category (105).

Validation should proceed in a stepwise manner: first, biological plausibility testing in patient-derived organoids, immune co-cultures, GBM-on-a-chip systems, and spatially resolved models (97, 100, 103, 104); second, retrospective and external validation in multicenter longitudinal cohorts with locked model parameters (74); and third, prospective silent-mode evaluation before any interventional or adaptive clinical use is considered (80, 85, 86). This staged approach is intended to prevent a conceptual model from being mistaken for an already validated decision system.

No single platform provides definitive ground truth (97, 98). However, convergence across complementary systems can materially strengthen the plausibility of inferred states and reduce the risk of conflating correlation with mechanism (59, 97). Biological validation is therefore best understood as a plausibility filter that links computational prediction to experimentally grounded interpretation before interventional use is considered (59, 97, 98).

### 5.2 Clinical feedback: from observation to adaptive refinement

Once biologic plausibility has been examined, the next step is clinical embedding without premature intervention. Serial imaging aligned with RANO 2.0 assessment points, liquid-biopsy kinetics, treatment exposure, corticosteroid use, neurologic status, and outcome data should be captured in a standardized manner so that model predictions can be compared with real patient trajectories under routine conditions (84, 106, 107).

These clinical observations are more than retrospective labels; they are feedback signals for adaptive refinement. If observed trajectories repeatedly diverge from predicted transition timing, pseudoprogression estimates, or reprogrammability scores, recalibration or constrained model revision may be warranted. Silent-mode evaluation is especially valuable because it enables real-time prediction generation without exposing patients to premature algorithm-guided decisions, while still revealing workflow fit, calibration, and concordance with multidisciplinary tumor board reasoning (74, 80).

This stage also requires a standardized minimum longitudinal dataset. At a practical minimum, that would include serial multiparametric MRI, perfusion measures, treatment milestones, corticosteroid exposure, neurologic status, and liquid-biopsy trajectories when available. Clinical feedback does more than score model performance; it also helps define how much longitudinal signal consistency is required before state inference can be considered clinically credible (22, 84, 107).

In a practical workflow, data collection would begin at surgery or postoperative baseline and continue at scheduled MRI assessments and clinically indicated visits. Imaging, treatment exposure, corticosteroid dose, neurologic status, molecular markers, and liquid-biopsy trajectories would be harmonized into a longitudinal data layer (11, 22, 23, 107). The model would generate phase probabilities, PsP/TuP probabilities, CTW-proximity estimates, reversibility estimates, and uncertainty scores before tumor board review (64, 74, 80). Outputs would remain decision support only, with multidisciplinary confirmation required before any clinical action; low-confidence outputs would trigger repeat imaging, biomarker reassessment, or tissue confirmation when feasible.

### 5.3 Regulatory control: governing adaptive AI systems

Because the GBM-ISFL is explicitly adaptive, it also requires a regulatory framework that allows controlled evolution without compromising safety (74, 85). The AI system should be positioned as clinician-facing decision support rather than autonomous decision-making, with final responsibility remaining with the physician and the multidisciplinary team (79, 85).

Update governance should follow predefined, reviewable rules. Permissible changes—such as recalibration, threshold adjustment, or updates within a validated architecture—should be specified in advance. Trigger criteria should likewise be explicit, including performance drift, dataset shift, or clinically meaningful changes in acquisition patterns. Any substantial update should undergo renewed external evaluation before release, and deployed systems should retain monitoring, auditability, version control, and rollback capability (74, 85, 86).

High uncertainty is both a clinical and regulatory boundary condition. When confidence is inadequate, the appropriate response is not automated escalation, but a hold-for-validation state that triggers repeat imaging, additional biomarker assessment, tissue confirmation when feasible, or multidisciplinary reassessment (78, 79).

Ethical implementation would also require explicit governance of patient privacy, informed consent for longitudinal multimodal data use, secure storage and de-identification, audit trails for model outputs, and transparent communication that the system provides decision support rather than autonomous treatment recommendations (85, 86). Patients and clinicians should be informed when model outputs are used in tumor board discussions, and model uncertainty should be displayed rather than hidden. Patient-specific safety considerations are especially important in immunotherapy because real-world data show that immune-related adverse events vary by clinical context, comorbidity, treatment regimen, and genetic susceptibility (108, 109).

With this combination of biologic validation, clinically embedded feedback, and lifecycle governance, the GBM-ISFL could be developed as a clinically accountable adaptive system rather than as a static predictive model (85, 97).

## 6 Constraints and translational boundaries of the GBM-ISFL framework

The GBM-ISFL is useful as an organizing framework, but it remains constrained by important biological, data-related, inferential, and translational limitations. Making those limitations explicit is essential. It prevents the framework from being overstated and distinguishes a clinically promising idea from a validated management system. The next two subsections outline these constraints and identify the conditions under which the framework can be prospectively tested—and potentially falsified.

### 6.1 Biological, data-related, and clinical constraints

At the biological level, GBM is profoundly heterogeneous across patients, across lesions, and even across regions within the same tumor. Any latent state vector inferred from imaging and liquid-biopsy data is therefore an approximation rather than a full reconstruction of the tumor–immune ecosystem. Rare but clinically important microstates—such as resistant invasive-margin clones or spatially restricted suppressive niches—may remain under-resolved even in strong models (9, 35, 110, 111). This is one reason why calibrated uncertainty and escalation to additional validation remain essential (78, 79).

Data quality and longitudinal consistency create a second constraint. The multimodal trajectories required for robust state inference are difficult to collect uniformly in real-world practice. Scanner protocols vary, follow-up intervals shift, and corticosteroid exposure, salvage therapy, and liquid-biopsy availability are uneven. Graph-based architectures may absorb some of this messiness, but they do not eliminate the need for harmonized acquisition, data-quality monitoring, and rigorous external testing (73, 74, 107).

Additional implementation barriers include computational cost, harmonization of imaging protocols, segmentation burden, delayed availability of liquid-biopsy results, limited access to high-quality longitudinal datasets, and the need for secure data infrastructure (73, 74, 107). Potential mitigations include standardized MRI acquisition, automated quality control, privacy-preserving or federated model development, modality-masking strategies, predefined uncertainty thresholds, and prospective workflow testing before clinical deployment (78, 79, 85, 86).

A related limitation concerns the quality of the labels used to supervise or benchmark these systems. Spatial transcriptomics, matched recurrent tissue, and serial immune profiling are available only in limited cohorts. Model targets may therefore be noisy, incomplete, or context dependent. Claims of mechanistic certainty should accordingly be restrained. Probabilistic state inference is not equivalent to direct biologic observation (15, 16, 110).

Clinical adoption introduces a further boundary. For decision support to be useful in neuro-oncology practice, clinicians must be able to examine why a model inferred a given phase or signal and to reject it when it conflicts with the broader clinical picture. A system that cannot be interrogated is unlikely to earn trust, regardless of how well it performs on paper (64, 79, 81).

There is also a straightforward translational reality: the framework has not yet been prospectively validated as an interventional system (64, 74, 80). At present, it should be regarded as hypothesis-generating and strategy-organizing rather than as a standard for patient management. Any move toward adaptive clinical use would require regulatory governance, predefined update boundaries, subgroup fairness assessment, and post-deployment surveillance (79, 85, 86).

These constraints do not invalidate the GBM-ISFL. Rather, they define the conditions under which adaptive, AI-supported longitudinal decision support could be developed responsibly, transparently, and with genuine clinical accountability.

### 6.2 Falsifiability and testable predictions of the GBM-ISFL framework

The value of the GBM-ISFL lies not only in its ability to organize existing evidence, but also in the explicit predictions it generates—predictions that can be prospectively tested and, if necessary, disproved. At present, those predictions should be understood as framework-level hypotheses rather than clinical rules.

One prediction is that entry into the Critical Transition Window should become detectable before unequivocal radiographic progression through convergent changes in imaging dynamics, perfusion measures, and liquid-biopsy trajectories. If no reproducible multimodal transition signal can be identified, the practical usefulness of the CTW concept would be substantially weakened (22, 23, 33).

A second prediction is that biologic phase should explain more variation in immunotherapy responsiveness than calendar time alone. The same therapeutic strategy should not be expected to behave identically before and after exhaustion locking, suppressive myeloid consolidation, or transition toward immune-desert conditions. If prospective studies fail to demonstrate such phase-dependent differences, the central phase-matching premise of the framework would be challenged (43, 47).

The framework also predicts that uncertainty-aware multimodal state inference could outperform fixed schedule-based heuristics and should therefore be tested for clinically important decisions, especially the distinction between pseudoprogression and true progression during the early post-radiotherapy interval. The benchmark is not discrimination alone, but also calibration, biologic coherence, and appropriate triggering of additional validation when uncertainty is high. If such systems cannot improve on the performance or practical utility of schedule-based assessment, their translational rationale becomes much weaker (33, 34, 77, 79).

A final prediction concerns reprogrammability. If that construct is biologically meaningful rather than merely rhetorical, states inferred to remain partly reversible should respond better to phase-matched reprogramming strategies than states inferred to be locked or dominated by immune-desert features. Failure to observe this relationship in experimental systems or prospective cohorts would argue against one of the framework’s central therapeutic assumptions (41, 52–54).

This matters because it provides the framework with a route to falsification as well as refinement. The GBM-ISFL should not be judged by conceptual neatness alone; it should be judged by whether its central claims survive prospective testing against longitudinal biology, clinically relevant decision points, and outcome-linked validation.

Taken together, these boundaries define the evidentiary and operational conditions under which the GBM-ISFL should be evaluated and further developed.

## 7 Discussion and future directions

This review presents the GBM Immune–Spatiotemporal Feedback Loop (GBM-ISFL) as a framework for rethinking immunotherapy in glioblastoma. It integrates a phase-based state-space atlas, noninvasive state inference, and phase-matched therapeutic logic into a single translational model. The central argument is straightforward: treatment failure in GBM may reflect not only inadequate agents, but also poor alignment between when therapy is delivered and the biologic state of the tumor–immune ecosystem.

The contribution is therefore conceptual rather than prescriptive. Biologic phase—not calendar time alone—is proposed as a major organizing variable for therapeutic reasoning in GBM (64). On that basis, this review defines a patient-specific Critical Transition Window, positions STGNNs as candidate tools for longitudinal state inference, and situates adaptive use within a framework of uncertainty management, external validation, and clinician-led governance.

The negative results of CheckMate-143, CheckMate-498, and CheckMate-548 should not be interpreted as direct evidence for the CTW hypothesis (5–7). These trials were not designed to test state-dependent treatment timing, longitudinal immune-state inference, or phase-matched intervention. Rather, they illustrate the limitations of fixed treatment assignment in a biologically heterogeneous disease where immune accessibility, myeloid dominance, corticosteroid exposure, MGMT status, recurrence setting, and treatment-related imaging changes may all influence response. Within the GBM-ISFL framework, future trials would require prospective stratification by longitudinal imaging and biomarker trajectories, predefined uncertainty thresholds, and adaptive trial designs that test whether state-informed enrollment or treatment timing improves biologic and clinical endpoints (5–7, 96). Future adaptive trials should also prospectively capture patient-level safety signals, because immune-related adverse events and ICI-associated acute kidney injury may vary across clinical context and genetic susceptibility (108, 109).

The most credible next steps are clear. Experimentally, patient-derived organoids, immune co-culture systems, GBM-on-a-chip platforms, and related models are needed to test whether inferred states and reprogrammability signals correspond to genuine biologic behavior (97–100). Additional microfluidic, three-dimensional, and tumor-organoid platforms can further extend this validation ecosystem (101–104). Clinically, standardized multimodal longitudinal cohorts and prospective silent-mode studies are needed to determine whether state inference improves pseudoprogression assessment, transition detection, and phase-matched therapeutic reasoning (106, 107).

If prospective validation supports these ideas, the GBM-ISFL could help shift GBM research from largely reactive salvage toward phase-matched, state-informed intervention. Until such validation is available, however, the framework should be used to organize hypotheses, design studies, and structure multidisciplinary reasoning rather than to direct routine patient management. More broadly, the same principles may prove relevant to other cancers and chronic diseases in which therapeutic timing depends on latent state transitions rather than fixed chronological milestones.
